# Pancreatitis, Panniculitis, and Polyarthritis Syndrome in Two Patients: A Case Series and Literature Review

**DOI:** 10.7759/cureus.59471

**Published:** 2024-05-01

**Authors:** Logan Kugathasan, Tony Zhuang, Justin Cheeley, Hope Khan, Audrey B Jernigan, Ketino Kobaidze

**Affiliations:** 1 Department of Medicine, Emory University School of Medicine, Atlanta, USA; 2 J. Willis Hurst Internal Medicine Residency Program, Emory University School of Medicine, Atlanta, USA; 3 Department of Dermatology, Emory University School of Medicine, Atlanta, USA; 4 Department of Nursing, Emory Healthcare, Atlanta, USA; 5 Department of Medicine, Division of Hospital Medicine, Emory University School of Medicine, Atlanta, USA

**Keywords:** pancreatitis panniculitis and polyarthritis syndrome, pancreatic cyst, ghost cells, polyarthritis, panniculitis, pancreatitis

## Abstract

Pancreatitis, panniculitis, and polyarthritis (PPP) syndrome presents a unique challenge in diagnosis and management because of its rarity and heterogeneous initial presentation. This manuscript presents a case series of two patients with PPP syndrome, shedding light on the diagnostic process and care for this uncommon condition. PPP syndrome is characterized by the simultaneous occurrence of pancreatitis or pseudocysts alongside polyarthritis and panniculitis. While its exact pathophysiology remains obscure, pancreatic inflammation is assumed to trigger the hematogenous dissemination of pancreatic enzymes, leading to fat necrosis and subsequent panniculitis, as well as chondronecrosis and/or osteonecrosis causing polyarthritis. Despite its recognition in medical literature since the late 1980s, PPP syndrome remains poorly understood, with only a limited number of cases reported globally. Its rarity and varied initial manifestations often result in misdiagnosis, causing delays in appropriate treatment. The presented case series highlights key clinical features and diagnostic clues of PPP syndrome. Both patients exhibited initial symptoms of inflammatory polyarthritis, accompanied by characteristic findings of "ghost cells" on skin biopsy. Additionally, radiographic and laboratory evidence revealed pancreatic changes consistent with this syndrome. This case series underscores the importance of multidisciplinary collaboration in managing PPP syndrome. Early recognition and accurate diagnosis are pivotal in initiating prompt and effective therapeutic interventions, thereby improving patient outcomes and minimizing long-term sequelae.

## Introduction

Pancreatitis, panniculitis, and polyarthritis (PPP) syndrome is an uncommon constellation of symptoms characterized by pancreatitis or pseudocysts with concomitant polyarthritis and panniculitis. While its pathophysiology is not well-known, it is theorized that inciting pancreatic inflammation and consequent hematogenous dissemination of pancreatic enzymes triggers fat necrosis, leading to panniculitis and chondronecrosis and/or osteonecrosis resulting in polyarthritis [1].

Because of its rarity, there is no well-established prevalence rate for PPP syndrome. The syndrome was first described in the medical literature in the late 1980s, and, since then, only a limited number of cases have been reported worldwide [2,3]. Because of its relative rarity and heterogeneous initial presentation, PPP syndrome can often be misdiagnosed, leading to delays in appropriate treatment.

This two-patient case series highlights the diagnosis and treatment of PPP syndrome. Both cases demonstrate initial presentations of inflammatory polyarthritis, findings of anucleate “ghost cells” on skin biopsy, and pancreatic changes both radiographically and on lab tests consistent with this condition. One case demonstrates an associated osteomyelitis, and the other demonstrates an associated reactive ileus, both conditions not traditionally associated with PPP syndrome.

## Case presentation

Case 1

**Figure 1:**
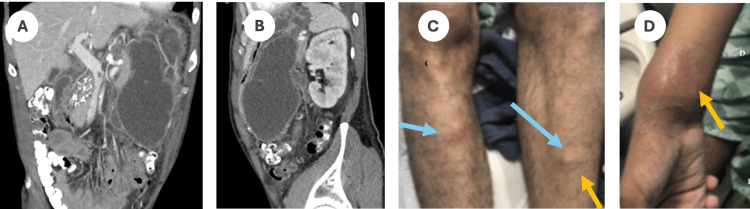
Case 1: (A) and (B) Presentation of a pancreatic pseudocyst. (C) Nodular erythematous, tender, ill-defined nodules on the shins. (D) Nodular erythematous, fluctuant nodule affecting the distal right forearm and wrist in the right distal forearm.

A 41-year-old man with a history of alcohol use disorder but no history of pancreatitis initially presented from a neighboring hospital for a two-week history of persistent, tender, bilateral ankle swelling. Initial treatment with antibiotics and steroids did not relieve his symptoms. On arrival, his temperature was elevated to 39°C. The patient reported mild epigastric pain and poor appetite, and he was not able to ambulate. A physical exam revealed tender ankle effusions, erythematous and tender nodules on the bilateral shins, right distal forearm swelling, and proximal interphalangeal joint swelling of the hands and feet (Figure 1).

Initial laboratory tests showed elevated C-reactive protein (CRP) of 245, erythrocyte sedimentation rate (ESR) of 67 mm/hour (reference range: 0-15 mm/hour), leukocytosis of 19,000 u/L (4,500-11,000 u/L), and elevated lipase of 231 u/L (0-160 u/L) (Table 1). Autoimmune and infectious workups were unremarkable. Because of the patient’s persistent fever and leukocytosis, the infectious disease team was consulted for concern of osteomyelitis, and the patient was initiated on vancomycin and ceftriaxone.

**Table 1:** Relevant laboratory data for Case 1.

Parameter	Result	Reference range
Blood chemistries		
Aspartate transaminase	13 U/L	13–39 U/L
Alanine transaminase	7 U/L	7–52 U/L
Alkaline phosphatase	67 U/L	34–104 U/L
Lipase	945 U/L	0–160 U/L
White blood cells	19,200 µ/L	4,500–11,000 µ/L
Erythrocyte sedimentation rate	63 mm/hour	0–15 mm/hour
Autoimmune panel		
Antinuclear antibody	Negative	Negative
Rheumatoid factor		
Double-stranded DNA		
Serum protein electrophoresis		
Infectious panel		
Gonorrhea/chlamydia/trichomoniasis	Negative	Negative
Antistreptolysin		
HIV		
Rapid plasma reagin		
Cryptococcal antigen		
Coccidioides		
Histoplasmosis		
Cultures		
Blood cultures	No growth	Sterile
Joint aspirate		

**Figure 2:**
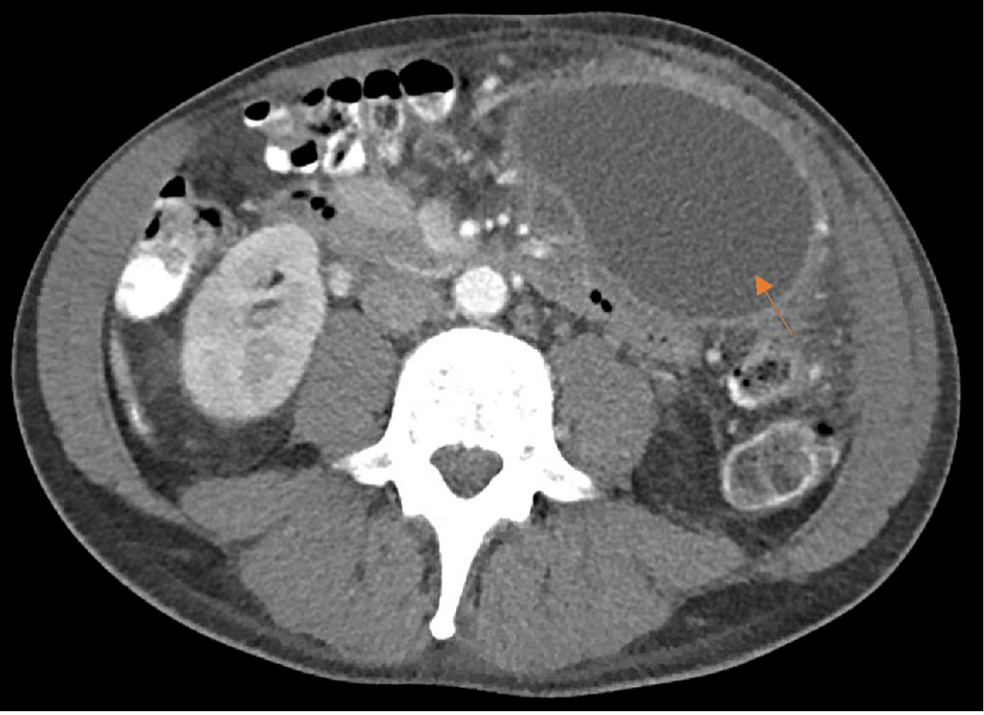
Case 1. Pancreatic pseudocyst measuring 17.0 x 7.7 x 7.5 cm abutting the pancreatic tail displacing the gastric body (orange arrow). Coarse calcifications in the pancreatic head/uncinate process with moderate peripancreatic stranding.

A CT of the abdomen revealed acute chronic pancreatitis with a 17.0-cm multiloculated pseudocyst in the gastric lesser sac with a nonocclusive thrombus in the portomesenteric confluence (Figure 2).

**Figure 3:**
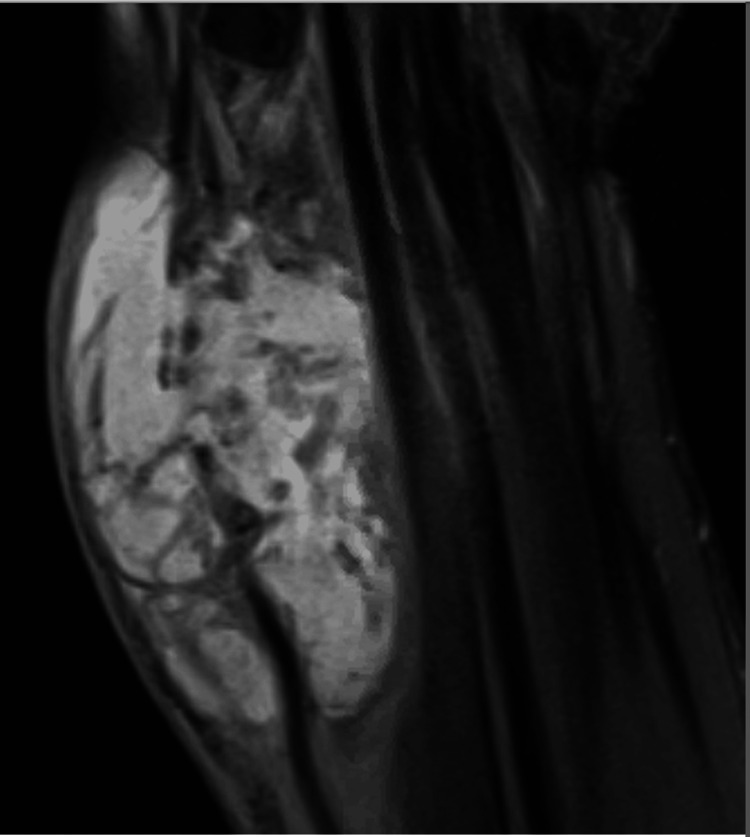
Case 1. 7.0 x 2.1 cm multiloculated complex fluid collection within the lateral aspect of the distal left forearm.

Gastroenterology, general surgery, and surgical oncology teams recommended observation with serial scans as the patient was too high risk for pancreatectomy and fistula repair. An MRI of the right wrist was significant for a 7 x 3 cm multiloculated, complex fluid collection with cortical erosions and irregularities encasing the flexor carpi radialis muscle and radial artery with severely heterogeneous marrow edema in the distal radius (Figure 3).

**Figure 4:**
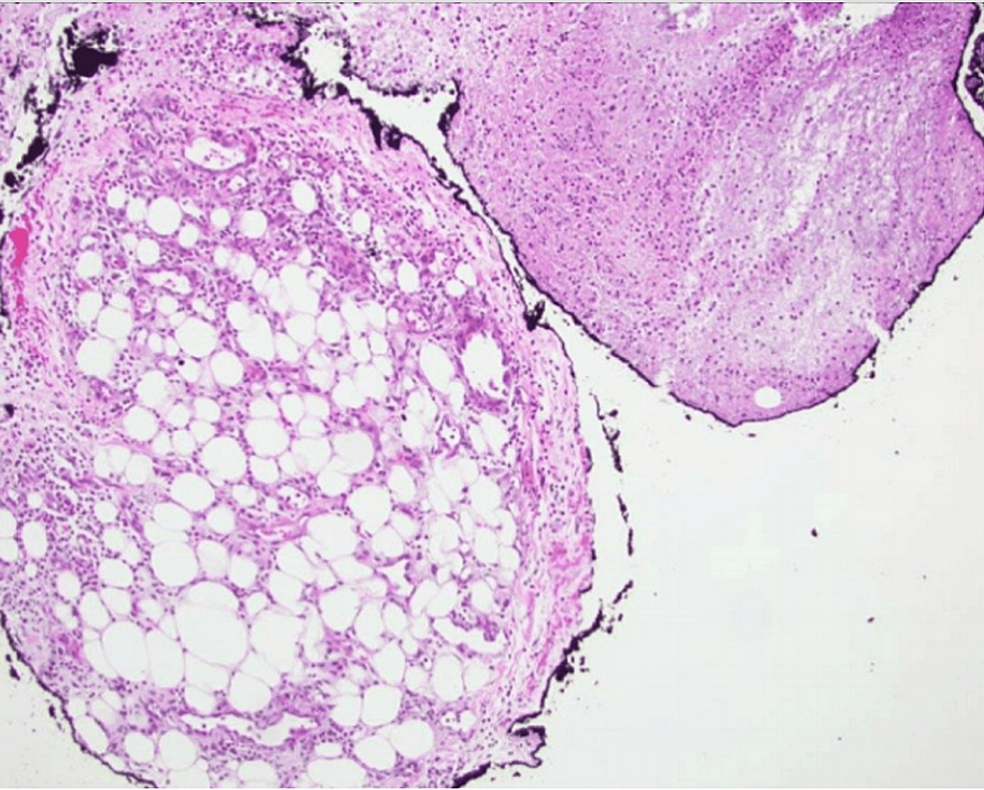
Case 1. Lobular panniculitis and anucleate and amorphous granular debris with surrounding basophilia and stippled calcification consistent with ghost cell histology consistent with pancreatic panniculitis.

Skin biopsy from a nodule on the shin revealed anucleate “ghost cells” in the adipose (Figure 4), consistent with pancreatic panniculitis. A left ankle arthrocentesis was performed, expressing a turbid, yellow fluid without neutrophils.

The patient was diagnosed with PPP syndrome and was treated with tapering glucocorticoids and a six-week course of vancomycin and ceftriaxone for concern of right wrist osteomyelitis. At the outpatient follow-up, the patient experienced an improvement in joint swelling, and he was able to keep up with daily living without assistance.

Case 2

A 48-year-old male with a history of alcohol-induced pancreatitis in the past presented to the emergency department (ED) for worsening right knee pain and swelling for one week. He was initially seen at a neighboring hospital one week prior for similar joint pains and bilateral shin nodules. The patient was diagnosed with erythema nodosum and discharged on prednisone and tramadol. During the previous hospitalization, his joint pain had spread to the bilateral knees and ankles. He had no abdominal pain.

**Figure 5:**
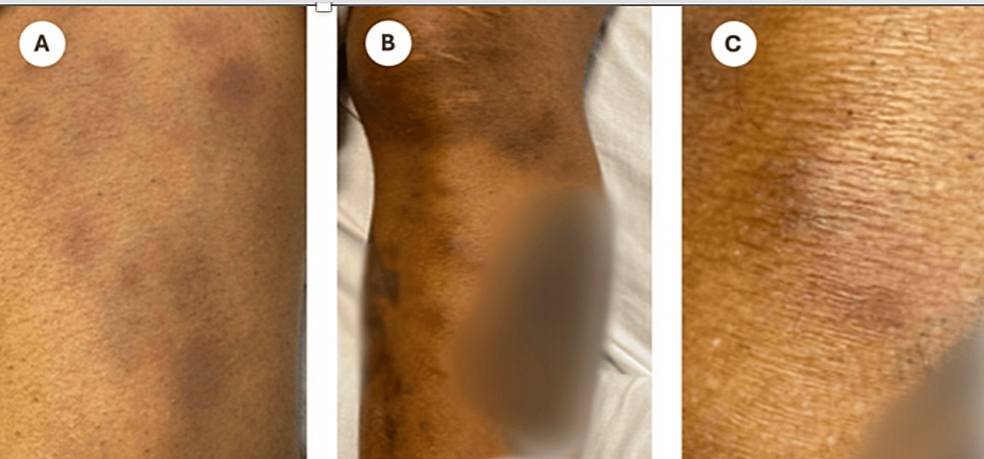
Case 2. Scattered, erythematous nodules on the bilateral thighs and shins. (A) Right anteromedial thigh, (B) left shin, and (C) close-up view of the left shin

On current ED arrival, he was afebrile, slightly hypertensive, and mildly tachycardic to 101 beats/min. He was unable to ambulate. A physical exam revealed bilateral leg pain, right knee effusion pain to palpation with a limited range of motion, and multiple nodules on the bilateral shins (Figure 5).

Laboratory tests were significant for elevated ESR of 91 mm/hour (0-15 mm/hour), elevated CRP 204.6, K 2.9, Na 135, leukocytosis at 21,400, and platelets at 488,000. Because of leukocytosis and initial monoarticular joint effusion in the absence of trauma, septic arthritis or gout was suspected, and the patient was administered intravenous vancomycin and ceftriaxone. Synovial fluid obtained through arthrocentesis of the right knee revealed positive monosodium urate crystals in only one of two fluid samples, low synovial white blood cell (WBC) count, and polyarthritis with reduced concern for gout. The patient was admitted for a broader workup of evolving polyarthritis with concern for autoimmune or infectious etiology. Rheumatology and infectious disease services were consulted. 

The autoimmune panel was negative (Table 2). Initial rapid plasma reagin (RPR) titer was positive at 1:16. Follow-up RPR was nonreactive, and anti-treponemal IgG/IgM was negative, suggesting an initial false-positive RPR. The remainder of the infectious workup was unremarkable except for a positive Chlamydia pneumoniae IgG, although C. pneumoniae did not fit with the patient’s clinical picture. A possible diagnosis of PPP syndrome was entertained considering the patient’s previous similar admission for acute pancreatitis with polyarthritis. Further workup detected elevated lipase over 3,000 u/L (0-160 u/L).

**Table 2:** Relevant laboratory data for Case 2.

Parameter (reference range)	Result	Reference range
Blood chemistries		
Aspartate transaminase	52 U/L	13–39 U/L
Alanine transaminase	25 U/L	7–52 U/L
Alkaline phosphatase	93 U/L	34–104 U/L
Lipase	>3,000 u/L	0–160 u/L
Leukocytes	21,400/µL	4,500–11,000/µL
Erythrocyte sedimentation rate	91 mm/hour	0–15 mm/hour
Autoimmune panel		
Antinuclear antibody	Negative unless stated	Negative
Rheumatoid factor		
Cyclic citrullinated peptide		
Double-stranded DNA		
Infectious panel		
Gonorrhea/chlamydia/trichomoniasis	Negative unless stated	Negative
Antistreptolysin		
Interferon gamma release assay		
C. pneumoniae IgG	Positive	Negative
HIV		
Syphilis IgM/IgG		
Cryptococcal antigen		
Coccidioides		
Histoplasmosis		
Hepatitis panel		
Ehrlichiosis/Borrelia		
Cultures		
Blood cultures	No growth	Sterile
Joint aspirate		

**Figure 6:**
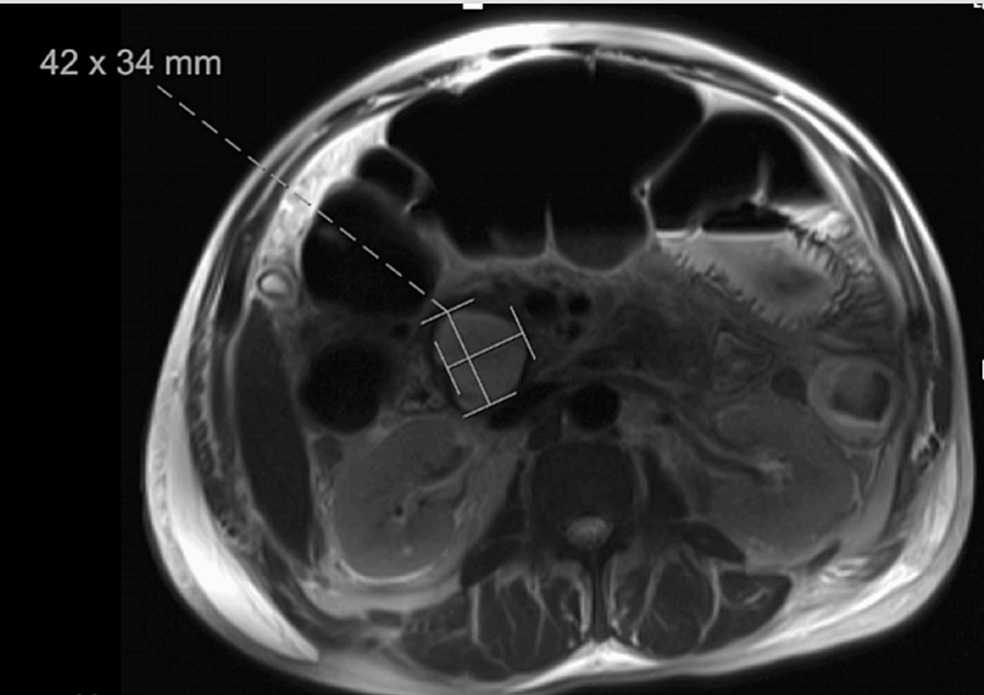
Case 2. An MRI revealing encapsulated complex fluid collection with rim enhancement measuring 4.2 x 3.4 cm, suggestive of a pancreatic pseudocyst without pancreatic ductal dilation or evidence of fistula.

CT abdomen showed a pancreatic pseudocyst of the pancreatic head, which was confirmed on MRI (Figure 6).

**Figure 7:**
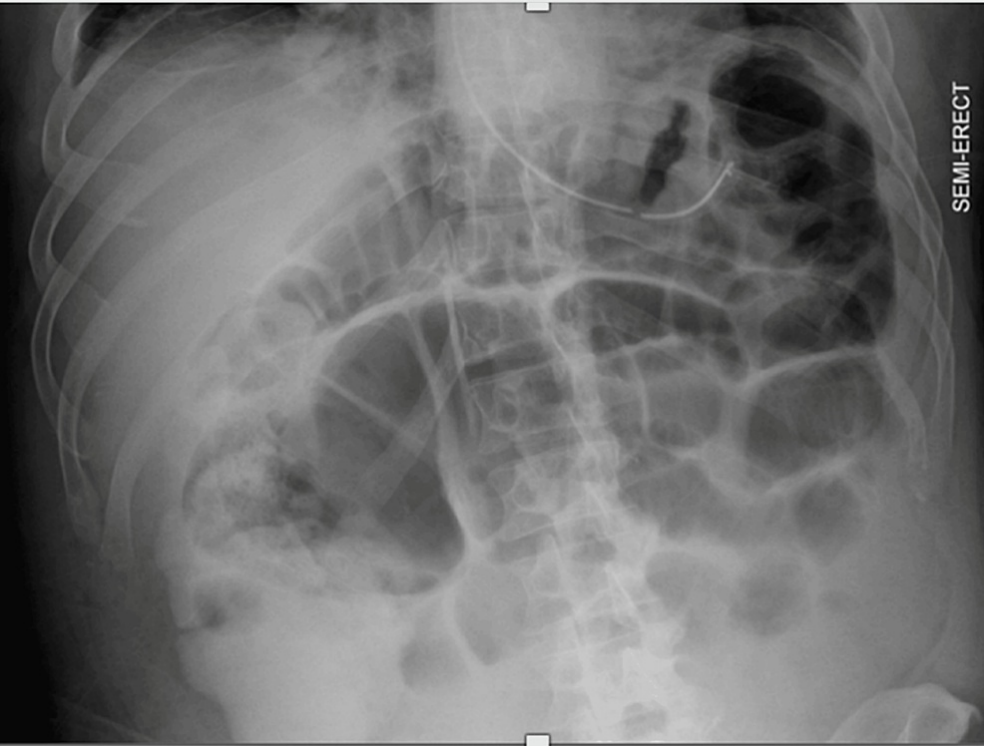
Case 2. X-ray of the thoracoabdominal high KUB revealing ongoing ileus.

Gastroenterology and general surgery teams recommended observation with supportive care. Serial abdominal kidney, ureter, and bladder (KUB) X-rays revealed ileus (Figure 7), correlating with the patient’s evolving diffuse abdominal pain and lack of bowel movements.

**Figure 8:**
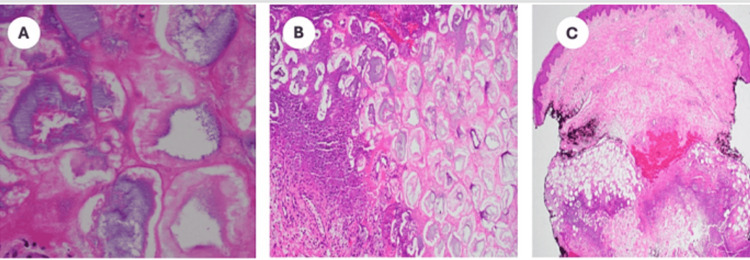
Case 2. Diffuse, interstitial neutrophilic infiltrate involving the subcutis with adipose "ghost cells" and associated calcification, H&E. (A) Original magnification 400x. (B) Original magnification 100x. (C) Original magnification 10px.

A punch biopsy of an erythematous nodule on the right medial thigh was performed, showing a neutrophilic infiltrate of the subcutis with the presence of adipose “ghost cells” (Figure 8). The patient required prolonged hospitalization for the management of reactive ileus and polyarticular edema with limited movement.

## Discussion

PPP syndrome is associated with acute or chronic pancreatitis, pancreatic pseudocysts, or pancreatic neoplasms as well as extra-pancreatic sequelae such as lobular panniculitis or polyarthritis. Any clinical finding within the triad may be associated with the initial presentation and can potentially confound medical decision-making. Owing to its complex pathophysiology and rarity, this condition requires early multidisciplinary discussion for appropriate evaluation and treatment.

An initial presentation of polyarthritis can mimic septic arthritis, gout, calcium pyrophosphate deposition disease, disseminated gonococcal infection, or rheumatoid arthritis [1,2]. Arthrocentesis can express viscous, noninflammatory joint fluid. Tender, erythematous nodules of the extremities can mimic other panniculitides, including infectious, reactive, and inflammatory processes. A skin biopsy revealing “ghost cells,” which represent anucleate adipocytes with thickened walls because of coagulative necrosis, is diagnostic of pancreatic panniculitis [2,3]. Abdominal symptoms are reported as mild or absent in almost two-thirds of patients, which may further contribute to misdiagnosis [4]. A history of abdominal trauma or alcohol use in the absence of abdominal symptoms should prompt consideration of this syndrome if other hepatobiliary, rheumatological, and infectious etiologies have been ruled out. A demonstration of elevated lipase enzymes or radiographic evidence of pancreatic inflammation can help guide the initial workup. Other pancreatic enzymes such as amylase may also be implicated, but amylase has been reported within normal limits in confirmed PPP syndrome [5]. Misdiagnosis or delays in accurate diagnosis can delay appropriate treatment, which may contribute to an alarming mortality rate of 24% [4].

While PPP syndrome is associated with bone marrow involvement and bony inflammatory reactive changes, there has only been one other case report detailing a concurrent finding of PPP syndrome with osteomyelitis, as found in our first patient [6]. A definitive etiology was not found to be associated with the patient’s presentation of his right wrist bony osteonecrosis, fever, and leukocytosis. Long-term insidious lipase-induced bony destruction may predispose to an infection. Regardless of association, a patient’s objective data should be considered in the context of clinical findings with a high degree of suspicion for secondary infection in PPP syndrome.

Of note, evidence of ileus (Figure 7) observed in the second patient has not previously been reported in PPP syndrome. While uncommon, ileus is a known complication of acute pancreatitis. It is possible that either ongoing severe inflammation of the body of the pancreas caused extrinsic compression or dissemination of pancreatic enzymes triggered a pericolitis [7]. Serial abdominal exams and plain films, bowel decompression, and bowel rest are indicated to monitor the progression of the ileus and determine whether surgical intervention is necessary.

Treatment of PPP syndrome often depends on the degree of pancreatic dysfunction. If a fistula into critical vasculature (often venous > arterial) with or without thrombosis is seen on cross-sectional imaging, prompt surgical consultation with fistulectomy and vascular repair may be indicated [2]. In the setting of pancreatitis or pancreatic pseudocyst without fistula, as reported in both patients, symptom management and observation may be warranted. Per prior case reports, patients have demonstrated symptomatic improvement in panniculitis and polyarthritis via oral prednisone taper and nonsteroidal anti-inflammatory drug treatment, although the chronic course of polyarthritis shows a poor response to these medications [4,8].

## Conclusions

PPP syndrome is a rare condition that requires a multidisciplinary approach for timely diagnosis and treatment. Early suspicion, workup, and appropriate treatment of PPP syndrome may improve prognosis. A finding of ghost cells on skin biopsy, acute changes in pancreatic enzymes or radiographic changes in the pancreas, and bony destruction should prompt consideration of this condition. The decision to pursue surgical intervention may depend on the level of pancreatic dysfunction and presence of fistula with or without thrombosis. Regardless, consultations with rheumatology, infectious disease, dermatology, surgery, and gastroenterology may be warranted for diagnosis and management. Any concurrently found condition that may or may not be associated with the diagnosis such as (but not limited to) osteomyelitis and reactive ileus should be seriously considered and treated as well.
